# Pediatric Patient with a Diagnosis of Pelvic Extraosseous Ewing’s Sarcoma: A Case Report

**DOI:** 10.5334/jbsr.3249

**Published:** 2023-09-07

**Authors:** Sabrina Amaouche, Christine Devalck, Nasroolla Damry

**Affiliations:** 1 Uz Brussel, Belgium; 2 Universitair Kinderziekenhuis Koningin Fabiola, Belgium; 3 Universitair Kinderziekenhuis Koningin Fabiola, CHU Brugmann, Belgium

**Keywords:** Ewing Sarcoma, pelvic region, rapid-growing, pelvic obstructive symptoms, pediatric, malignant tumor

## Abstract

Ewing’s sarcoma (ES) is a malignant tumor that arises mainly from bone tissue. Primary extraosseous Ewing sarcoma (EES) is a rare form of the Ewing’s sarcoma family of tumor, and pelvic localization is even more unusual, considered to be one of the rarest localizations [1]. We present the case of a seven-year-old boy with persistent abdominal pain. Ultrasound (US), contrast-enhanced computed tomography (CECT), and magnetic resonance imaging (MRI) revealed the presence of a large, solid, and heterogeneous mass in the pelvis. The histological and immunohistochemistry were compatible with pelvic EES.

**Teaching point:** Extraosseous Ewing’s sarcoma is a rare pediatric tumoral entity that requires clinician and radiological vigilance and detection.

## Introduction

Ewing’s sarcoma (ES) is a highly malignant tumor, considered the second most common primary bone malignancy in the pediatric population [2]. It can arise from bone or soft tissue. It is classified into four types according to the origin of the tumor: Ewing’s sarcoma of the bone, peripheral primitive neuroectodermal tumor (pPNET), Askin tumor, and extraosseous Ewing’s sarcoma (EES).

EES represents 10–20% of cases of the ES family [3], and pelvic localization is uncommon; there are only a few cases reported in the literature on pelvic EES.

## Case History

A seven-year-old boy presented to the emergency department with worsening pain in the lower abdomen for one week and frequent voiding with small amounts of urine. On abdominal examination, a hard mass measuring approximately 7 cm in the right iliac fossa was palpated.

**Figure 1:**
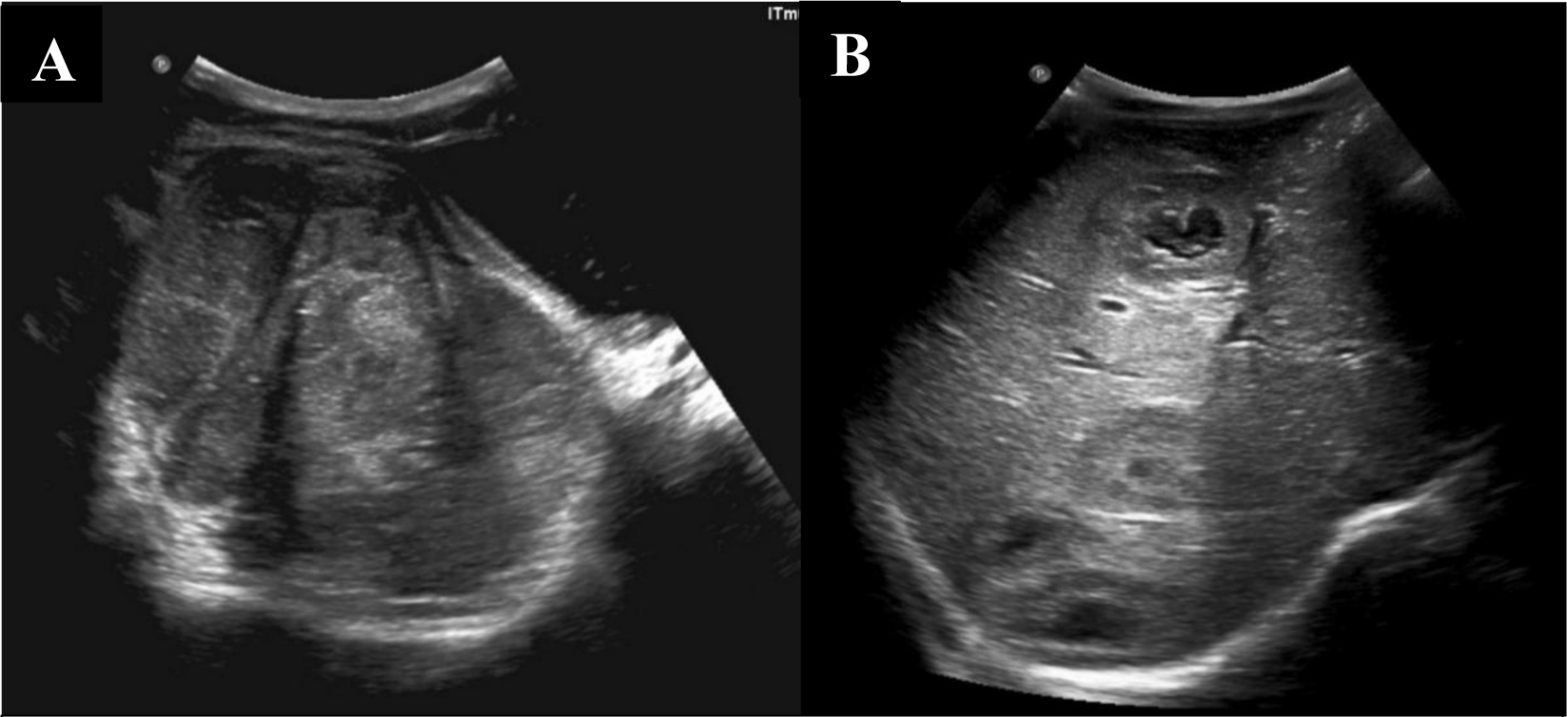
US images of a seven-year-old boy with pelvic extraosseous Ewing’s sarcoma. Image **(A)** shows low echogenicity pelvic soft tissue mass. Image **(B)** shows several solid nodular hepatic lesions with a bull’s-eye appearance suggestive of liver metastases.

Ultrasound (US) revealed a heterogeneous mass of low echogenicity (Figure 1A) as well as several solid nodular hepatic lesions with a bull’s-eye appearance suggestive of metastases (Figure 1B). Color Doppler revealed arterial and venous blood vessels within the pelvic mass.

**Figure 2:**
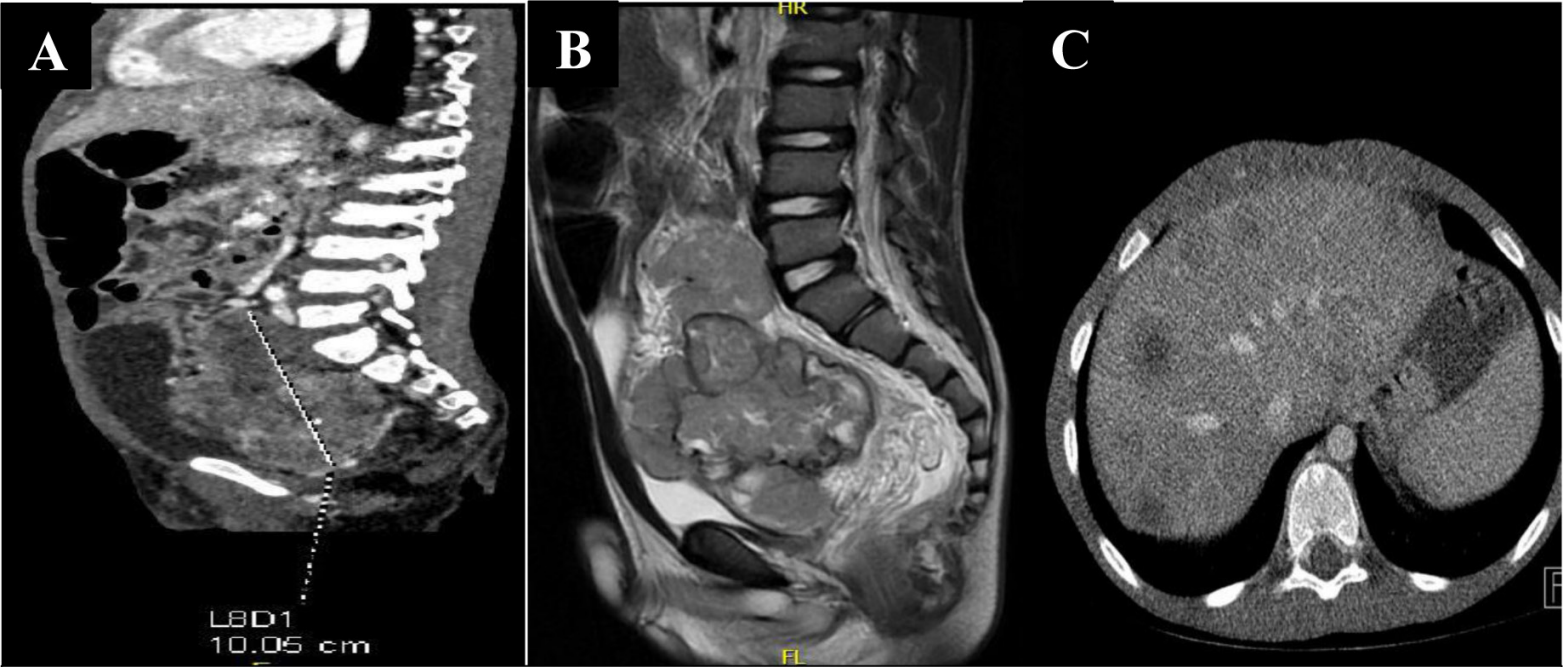
Sagittal CT image **(A)** shows a large mass with heterogeneous enhancement in the portal phase measuring 10 cm in maximum diameter, occupying almost the entire pelvis and extending up to L4–L5. Sagittal T2-weighted MRI **(B)** shows a large pelvic mass that correlates with the CT image. Axial CT image **(C)** confirms liver metastases.

Contrast-enhanced computed tomography (CECT) scan confirmed the presence of this large mass with heterogeneous enhancement in the portal phase measuring 10 cm in maximum diameter and occupying almost the entire pelvis and extending up to L4–L5 (Figure 2A). CECT confirmed also liver metastasis (Figure 2C). Sagittal T2-weighted magnetic resonance imaging (MRI) correlated well with the computed tomography (CT) findings (Figure 2B).

**Figure 3:**
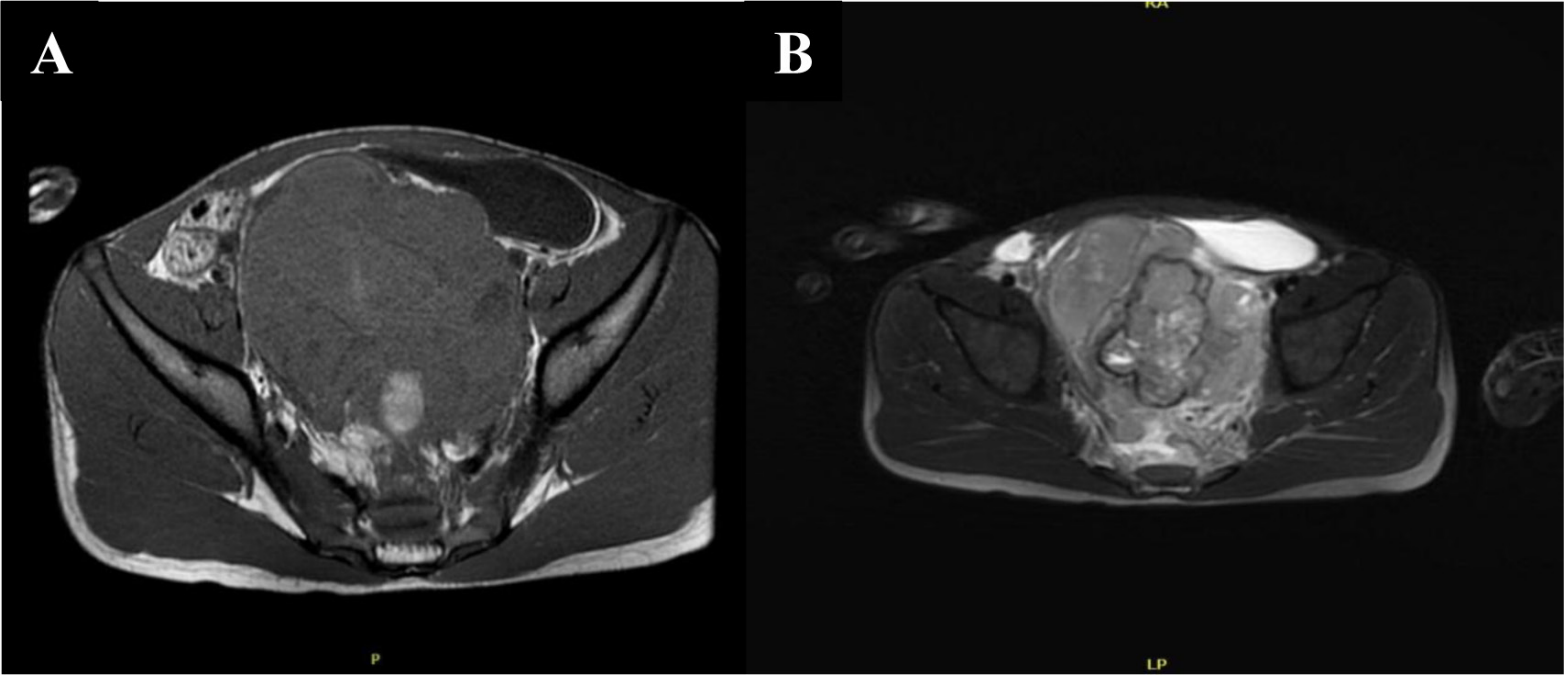
Axial T1-weighted MRI image **(A)** shows a well-defined, heterogeneous low signal intensity and lobulated pelvic mass with focal hyperintensies indicating hemorrhagic zones. Axial T2-weighted image **(B)** shows a hyperintense and heterogeneous content.

MRI showed a large, well-defined, lobulated and heterogeneous pelvic mass hypointense on T1 with persisting focal hyperintensies when fat is saturated, suggesting hemorrhagic areas (Figure 3A). On T2-weighted images, the mass showed hyperintense and heterogeneous content (Figure 3B).

**Figure 4:**
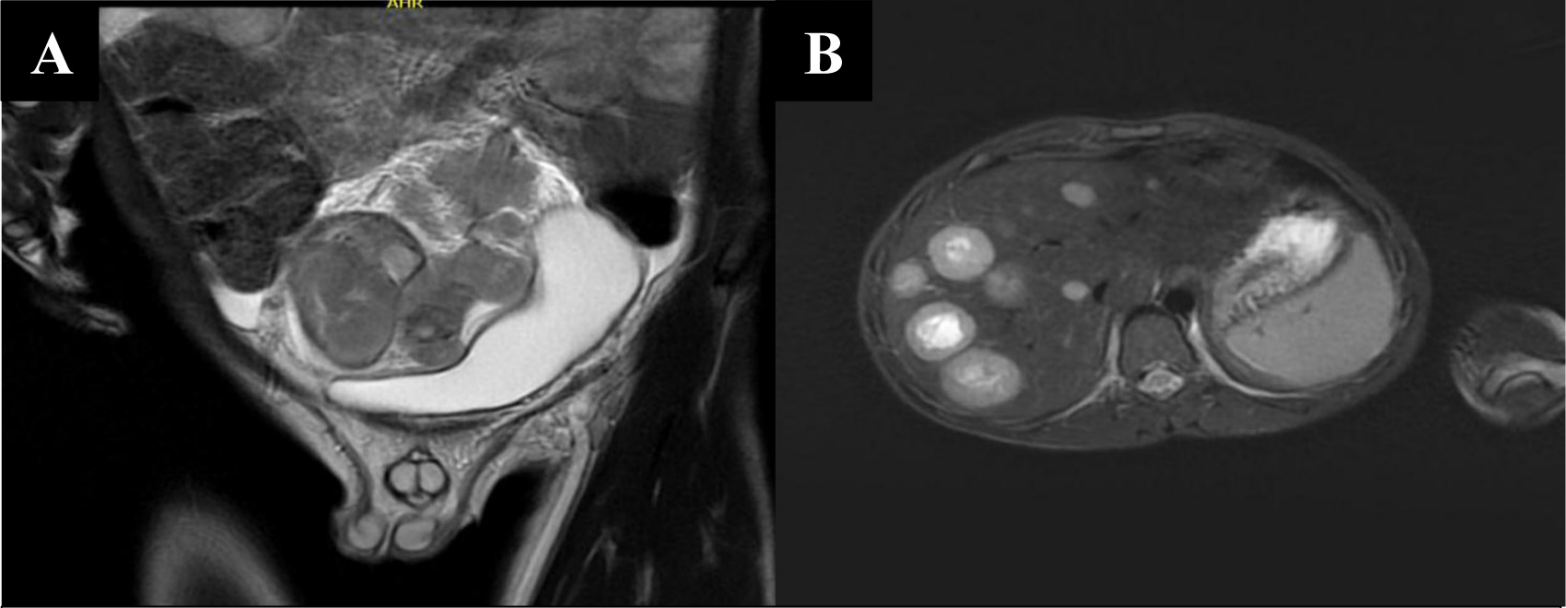
Coronal T2-weighted MRI image **(A)** shows bladder compression. Axial T2-weighted MRI image **(B)** shows multiple hepatic metastases with central necrosis.

This lesion had a slight contact with the rectum and the lumbar spine, but without any signs of invasion. The bladder was compressed, which explains the urinary symptoms (Figure 4A). MRI also confirmed hepatic metastases with central necrosis (Figure 4B).

The patient underwent an incisional biopsy and the pathological findings microscopically revealed the presence of a small round blue cell tumor and the immunohistochemistry was compatible with ES.

There was no ESWRI (ES) or FKHR (RMS) rearrangement.

The patient was referred to the surgical oncology department for chemotherapy and surgery.

## Comments

Pelvic ESS is considered to be one of the rarest locations of EES in children. Symptoms depend on the local mass effect of the tumor, such as oliguria following compression of the bladder or constipation following compression of the digestive tract.

Pelvic EES has no specific radiological characteristics; the final diagnosis of pelvic EES is confirmed by characteristic features on histologic analysis, histochemistry, and Immunohistochemistry [4,5].

Treatment of EES is multimodal with chemotherapy, surgery, and radiotherapy.

Pelvic EES has a poorer prognosis when compared to other sites, with 30%–40% of metastases at the time of diagnosis [6].

## Conclusion

Pelvic EES is a rapidly growing tumor with no specific radiological features and a poor prognosis compared with ES of the bone. It is therefore important for clinicians to recognize this entity, as early diagnosis and treatment help to improve prognosis.
